# Effects of competitive learning tools on medical students: A case study

**DOI:** 10.1371/journal.pone.0194096

**Published:** 2018-03-08

**Authors:** Alfredo Corell, Luisa M. Regueras, Elena Verdú, María J. Verdú, Juan P. de Castro

**Affiliations:** 1 School of Medicine, Universidad de Valladolid, Valladolid, Spain; 2 Higher Technical School of Telecommunications Engineering (ETSIT), Universidad de Valladolid, Valladolid, Spain; 3 School of Engineering and Technology, Universidad Internacional de La Rioja, Logroño, Spain; Waseda University, JAPAN

## Abstract

**Objective:**

Competitive learning techniques are being successfully used in courses of different disciplines. However, there is still a significant gap in analyzing their effects in medical students competing individually. The authors conducted this study to assess the effectiveness of the use of a competitive learning tool on the academic achievement and satisfaction of medical students.

**Methods:**

The authors collected data from a Human Immunology course in medical students (n = 285) and conducted a nonrandomized (quasi-experimental) control group pretest-posttest design. They used the Mann-Whitney U-test to measure the strength of the association between two variables and to compare the two student groups.

**Results:**

The improvement and academic outcomes of the experimental group students were significantly higher than those of the control group students. The students using the competitive learning tool had better academic performance, and they were satisfied with this type of learning. The study, however, had some limitations. The authors did not make a random assignment to the control and experimental groups and the groups were not completely homogenous.

**Conclusion:**

The use of competitive learning techniques motivates medical students, improves their academic outcomes and may foster the cooperation among students and provide a pleasant classroom environment. The authors are planning further studies with a more complete evaluation of cognitive learning styles or incorporating chronometry as well as team-competition.

## Introduction

In the last years, medical education has adopted active learning models to a progressive adaptation to the requirements of the Bologna Process. The current scenario of extensive use of computers and the Internet has created opportunities to improve education by applying different e-learning techniques in courses [1, 2]. Consequently, blended learning is replacing traditional face-to-face education and learning spaces (both virtual and physical) are being redesigned to support emerging strategies such as flipped classroom or gamification. In this context, motivating students to participate actively is just as important as improving their academic outcomes. Therefore, teachers need to understand the new available e-learning methods to apply them creatively and effectively [3].

There are a variety of learning strategies that can be introduced to engage students and to promote their critical thinking and deeper understanding. The effectiveness of methods such as cooperative learning, Problem-Based Learning (PBL) or Case-Based Learning (CBL) has been widely studied. CBL has been used in different medical schools as an alternative to traditional education with the aim of students to collaborate in small groups [4, 5]. Moreover, there are many studies that analyze the effectiveness of PBL in medical education. Dochy et al. [6] conducted a meta-analysis with forty-three articles whose results showed that there is a positive effect on students’ skills but not on knowledge acquisition, which is agreed with the review of the literature done by Colliver [7]. This study revealed that there is not convincing evidence about the improvement of knowledge and clinical performance with the use of PBL. In any case, Albanese and Mitchell [8] have stated that “even if knowledge acquisition and clinical skills are not improved by PBL, enhancing the work environment for students and faculty is a worthwhile goal in and of itself”. This is consistent with the finding that PBL provides a more enjoyable and motivating educational process for both students and faculty than traditional classes [9].

Like cooperative learning, competitive learning is another effective method to increase students’ motivation and satisfaction as well as to improve their learning achievements [10, 11]. Competitive learning has characteristics that lead to a greater engagement of students by arousing their competitive instincts [12]. Furthermore, according to the study done by Lempp [13], competitiveness is a characteristic of medical environment and schools. Competitive learning was usually associated with the traditional classroom and students’ competitive behavior, being object of a lot of criticism [14]. Nowadays, although the subject of much debate remains, competitive learning is becoming a powerful tool for blended and on-line learning environments, with digital or computer-based games as the best example. Even those researchers who had shown that competition had no effect on the students’ motivation, found relation between competition and student’s post-experimental perceived competence, interest and task value [15].

Competitive learning has been applied and analyzed in different disciplines [15, 16] and educational levels [17, 18]. There are also some studies about its use in medical schools. For example, Lei et al. [19] analyzed the effectiveness of introducing competition in a cooperative learning environment, based on CBL. The experiment was deployed in a clinical course about severe infection with 71 students. They observed that introducing team-competition improved the effectiveness of the teaching. The students with competition had better performance than the students only with CBL. On the other hand, Janssen et al. [20] assessed the perceived value by students of anatomy through surveys and semi-structured interviews with sixteen voluntary students. The students indicated that they particularly enjoyed the competitive aspect of the game. These studies had some limitations. Lei et al. [19] introduced an award in the competition mode to improve the teaching efficacy. Although they stated that the winners were more motivated by the sense of achievements than by the award, there was no evidence of that. The improvement in performance could not be a direct consequence of the competitive learning. Janssen et al. [20] carried out an experiment with very few students. Besides, they connected the engagement of students especially with the work in teams, more than with the competition itself, although a summative effect of both motivations could not be ruled out.

We can see other interesting findings in the study of Van Nulan et al. [21]. They assessed the effects of anonymous on-line competition on the academic performance of anatomical students. They found that the students who participated in an online tournament achieved higher performance than their noncompeting peers. They also demonstrated positive results about motivation and engagement. However, their research had some important limitations: participant attrition and selection bias. Although they recruited 67 voluntary students at the beginning of the experiment for participating in the game, more than half abandoned it. In fact, all the students who abandoned the game were assigned post-hoc to the control group. They did not neutralize this critical selection bias by assessing cognitive learning style preferences or learning anxiety, for example.

In summary, there is still a significant gap in analyzing the effects of on-line competitive learning in courses with a high number of students competing individually.

Therefore, the purpose of this study was to examine the use of a competitive learning methodology in an immunology course to assess both the effectiveness and the students’ satisfaction level with this type of learning, as well as the relationship between them. As we will describe later, the competitive tool applied in this experience integrates some interesting scoring and timing aspects, which are not present in the tools used in the previous studies reported above [19–21]. This combination is a better measurement of performance in those medical tasks in which the response time is so important [22].

The two hypotheses of the research were the following:

**H1**. The final grading scores of the students who use the competitive learning tool are higher than those of the students who do not use it.
**H2**. The students who use the competitive learning tool improve more their score than the students who do not use it.

## Methods

### Selection and description of participants

We implemented a quasi-experimental control group pretest-posttest design in a course of Human Immunology given at the Medical School of the University of Valladolid (Spain). This is a compulsory subject in the second year of the Degree in Medicine. The population consisted of 285 students, 71 men (24.91%) and 214 women (75.09%), with an average age of 19.94 years. All students were over 18 years of age and gave their verbal consent to participate in this study. Their anonymity was always preserved in the study by removing all personal identifiers from the data. As it is standard in socio-economic experiments, no ethic concerns were involved other than preserving the anonymity of participants. Moreover, we did not collect any sensitive data such as racial origin, religious beliefs or data concerning health (according to the Spanish Law for Personal Data Protection).

We wanted to analyze the students’ academic outcomes to study the effect of a competitive learning strategy on medical students. Therefore, we should establish experimental and control groups. However, the teacher decided not to divide their students randomly into two groups, because he felt it to be unfair for those students falling in the control group, and a completely randomized design was not possible. Therefore, the students chose if they would like to follow the itinerary A (control group) or the itinerary B (experimental group), where a competitive learning methodology would be used.

Finally, since we did not establish the groups randomly, we needed to verify that the two groups were similar and that they were not significantly different in other relevant aspects. Then, we did several previous studies based on the test of independence to check if factors such as the knowledge level or learning style had affected to the formation of the groups and thus, they could influence the results obtained.

### Competitive learning tool and strategy

**Fig 1:**
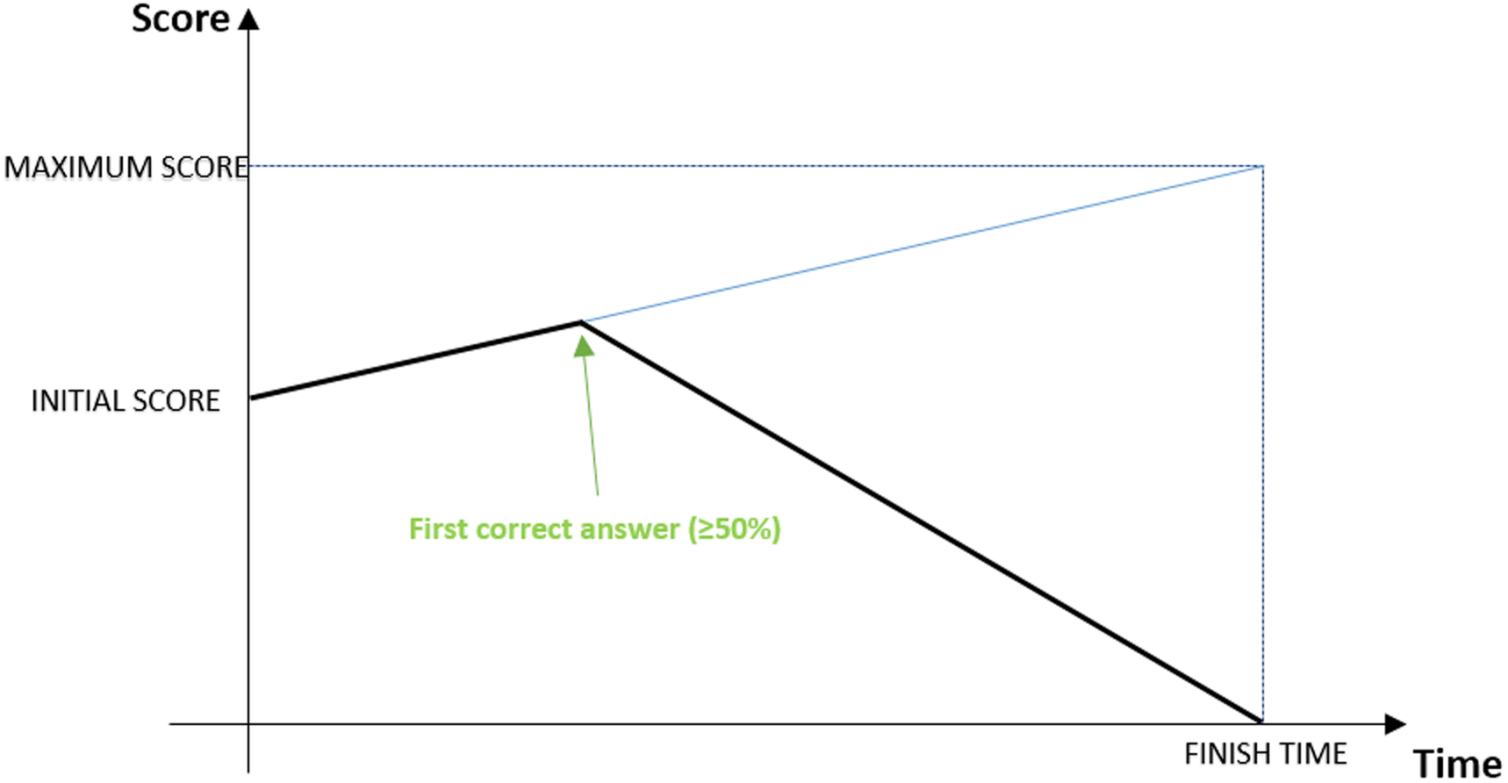
QUESTOURnament variable scoring system. The figure shows how the score varies. From the initial score, the variable score lineally increases until the first correct answer (≥50%) is received; then, the score starts decreasing until the minimum score (0 points), which should be reached at the ending time of the challenge.

We conducted the study by using a competitive e-learning tool called QUESTOURnament. This tool is a Moodle module that allows teachers to organize contests. Each contest includes a set of intellectual challenges or questions that students must solve in a time-constrained way. Students obtain points both by answering the proposed challenges and by submitting new questions and assessing the answers submitted by their classmates. The answers are scored like is shown in Fig 1. This variable scoring system depends on the initial score, the maximum score and the duration of the challenge set by the teacher. Initially, the score lineally increases until the first correct answer is received and evaluated; once a challenge is correctly answered, the score starts decreasing so that the student who answers first can get the maximum score [23].

QUESTOURnament differs from other competitive systems, such as the one used by Van Nulan et al. [21], in two main aspects:

- The students, besides answering questions, can propose new challenges to be solved by their peers and assess the received answers.
- Answers are scored according to the variable scoring system described above, instead other typical scoring systems, in which, for example, a binary correct/incorrect is combined with additional points to award the fastest answer [21].

**Fig 2:**
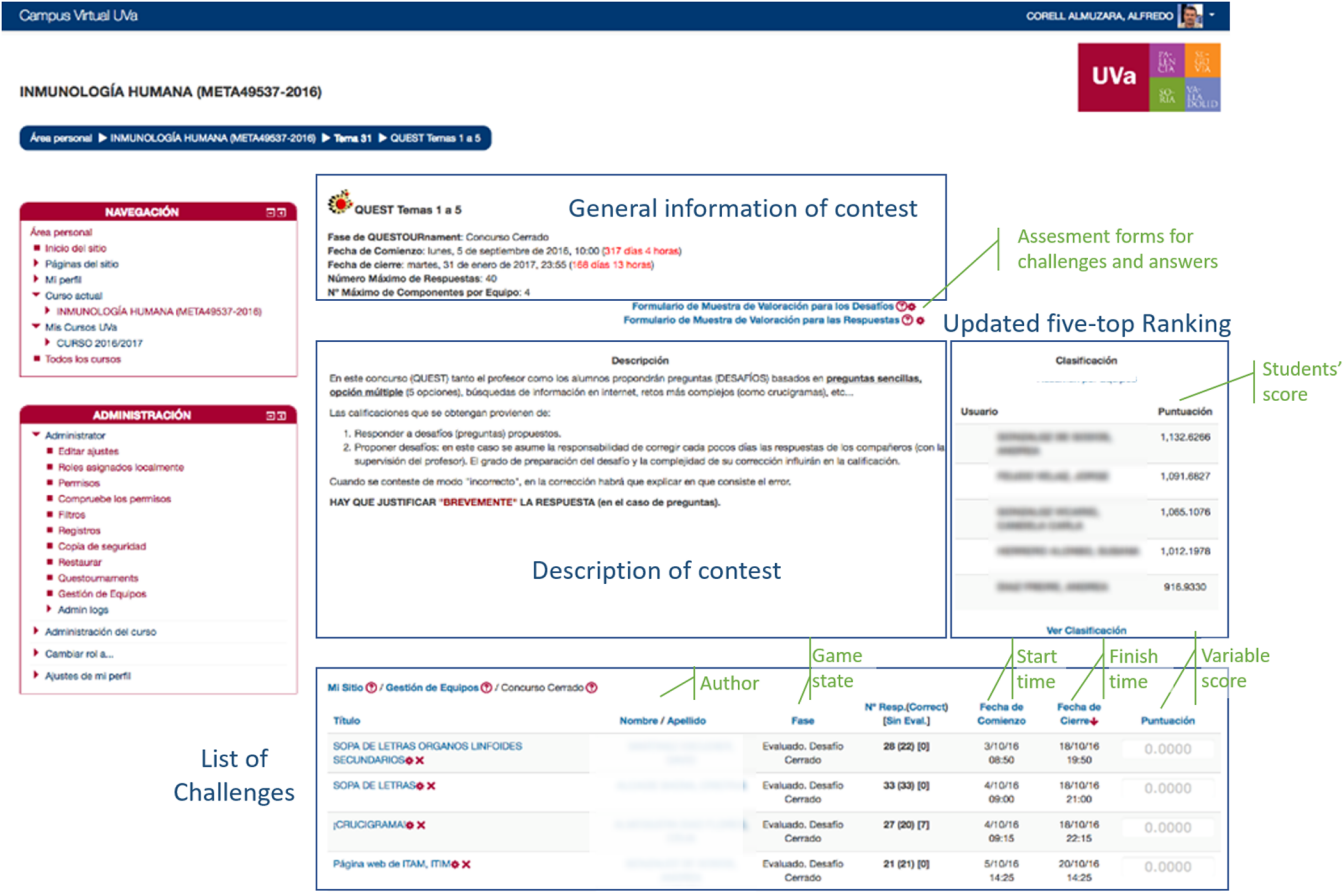
The QUESTOURnament tool in the Human Immunology course. The QUESTOURnament system permanently displays both an updated five-top ranking and the list of questions with their details (times, current score and game state-open or closed). General information and detailed description of the contest is also displayed as well as links to the assessment forms for challenges and answers.

The QUESTOURnament system permanently displays both an updated five-top ranking and the variable score of questions (see Fig 2), resulting in a dynamic environment that tries to promote students’ motivation and participation.

In this context, we organized the experience (itinerary B) according to the following aspects:

- Use of an organized e-learning course inserted in a Moodle-based virtual campus.
- The contests were individual and non-anonymous.
- There were four QUESTOURnament contests during the semester. Each student in the “itinerary B” should participate in at least two of the tournaments by means of:
  - proposing at least four challenges (two in each contest) and evaluating the answers given by their classmates, with teacher supervision.
  - answering to at least eight challenges proposed by other students.
- The challenges were diverse: multiple-choice questions, simple questions, Internet search queries, elaborate questions (such as crossword puzzles, word searches, etc.)
- The challenges lasted for fifteen days.

The teacher evaluated all challenges proposed by students according to an assessment form known by students at the time of proposing their challenges. The assessment criteria grid was simple. The challenges were evaluated in terms of their relevance to the Human Immunology topic (weighting factor of 2/7), the unambiguous redaction (weighting factor of 1/7) and, finally, in terms of creativity (weighting factor of 4/7). The weighted grades pursued to reward the most time-consuming and creative challenges.

On the other hand, there was also an assessment form for the answers, with two elements: correct or incorrect answer (weighting factor of 1/3) and quality of answer (weighting factor of 2/3). Students could also enter the scores manually as percentages (out of 100 points).

This learning strategy was very time consuming for the teacher. On the one hand, there was an initial practice session where students learn how to propose and assess a challenge with the QUESTOURnament tool. On the other hand, the teacher had to supervise, approve, refine and evaluate all the challenges proposed by the students; this could take about 5–10 minutes each (depending on the complexity of the proposal). Afterwards, the assessments of answers were done by the students. The teacher did not reevaluate these assessments, unless a problem was reported by any disappointed student (which happened very few times in this experiment, approximately one in 100).

The students of itinerary A followed the traditional class. They were free to use the Virtual Campus (where slides and notes were available), while the students of itinerary B compulsorily had to use it combined with the competitive learning tool QUESTOURnament. The grade obtained by students of itinerary B in QUESTOURnament activities supposed a minimum effect in the students’ final score, since it only accounted for 5% of the final score.

### Methodology and instruments

We used SPSS Statistics 20 for statistical analysis, employing the Mann-Whitney U-test for paired comparisons to measure the strength of the association between two variables.

We used two instruments in this study:

1. the students’ final grades in the target course (post-test) and in three different and previous courses (pre-test), which measured the knowledge level (on a scale of 10). To verify the effectiveness of competitive learning, this quasi-experimental study design combined the grade obtained in the immunology course with the mark obtained in three courses of the previous year (the first year of the degree). These three courses (Biochemistry, Genetics and Cellular Biology) are directly related to the Human Immunology course, because a deep knowledge of the three is considered necessary to understand the biochemical, cellular and genetic peculiarities of the immune system. Therefore, we considered them suitable as pre-tests for the quasi-experiment design.
2. a survey, which measured the students’ satisfaction and collected other interesting students’ data (S1 Appendix). The first part of the survey was a questionnaire about the students’ age and gender, learning style (independent, competitive, collaborative and contributory), level of class attendance and if they define themselves as hard-workers. The second part was a ten-item survey based on the method developed by Bures et al. [24], which measures students’ satisfaction and motivation in e-learning environments. It provides the students with a five-score Likert-type scale, which ranges from “Strongly Disagree” to “Strongly Agree” (1 to 5, respectively). The students’ satisfaction total score, generated by summing up all the scores, can range from 10 (very low satisfaction level) to 50 (very high satisfaction level).

## Results

Firstly, given that distribution of groups was not random, we needed to verify that the distribution of the two groups did not influence the results. Table 1 shows the students’ general data. We observed that the proportion of men enrolling within the itinerary B was slightly higher than among women (58% of women versus 66% of men). This observation was concordant to Chaput de Saintonge and Dunn [25], who established that men feel more motivated than women with competitive learning environments. Moreover, the students defined themselves as low competitive. Budakoglu et al. [26] and Grasha [27] supported this observation. They also found that competitive learning style scores were lower than other styles.

**Table 1:** Students’ data.

		Experimental group *(n = 172)*	Control group *(n = 113)*
**Sex**	Women	125	89
	Men	47	24
**Age**	<19 years	1	1
	19–20 years	129	102
	21–22 years	28	8
	>23 years	14	2
**Profile**	Competitive	14	3
	Collaborative	85	37
	Independent	99	71
	Contributive	35	24

We considered important to analyze if the two groups were not significantly different in other relevant aspects (level of prior knowledge, class attendance style or self-awareness as hard-workers). According to data of Table 2, we established the following results:

- The students of the experimental group had not the best scores in previous courses. Our results showed that the students of the control group had a slightly higher previous knowledge level in Biochemistry, Genetics and Cellular Biology, and moreover, this difference was significant (p < 0.005). Although we were trying to demonstrate the homogeneity of the two groups, this result was not a hindrance for studying the effects of the competitive strategy, since the students who followed it had a worse previous knowledge level (and thus, this difference was working against our assumption that the competitive itinerary students were the best students).
- The students of the experimental group were not the more laborious students. The results showed that the difference between the two groups was not significant (p > 0.05).
- The students of the experimental group were not who attend classes more. The results showed that the difference between the two groups was not significant (p > 0.05).

**Table 2:** Students’ characteristics: Experimental vs. control group.

	Control group (n = 113)		Experimental group (n = 172)		U Mann-Whitney	
	Mean	SD	Mean	SD	U	p
Knowledge level	5.82	1.85	5.28	1.93	-2.912	0.004*
Hard-working level	6.10	2.04	5.98	2.20	-0.234	0.407
Class attendance level	8.17	2.81	8.21	2.87	-0.504	0.307

* Results are significantly different at p < 0.005.

Once validated the formation of the experimental and control groups, we analyzed the students’ outcomes using the Mann-Whitney U-test to validate the two research hypotheses. The results, presented in Table 3, showed that the students’ final score grades in Human Immunology were significantly different between the two groups (p < 0.001), indicating that the hypothesis H1 was supported as the outcomes of the experimental group students were significantly higher than those of the control group students. Moreover, the improvement obtained in Human Immunology (when compared to highly related courses grades obtained in the first year) was significantly different between the two groups (p < 0.001), what could indicate that the hypothesis H2 was also supported. However, since the students who followed the experimental itinerary had a worse previous knowledge level, their margin of potential improvement was higher. Therefore, we could not state that the greater improvement was only due to the use of the competitive system.

**Table 3:** Score grades and improvement: Experimental vs. control group.

	Control group (n = 113)		Experimental group (n = 172)		U Mann-Whitney	
	Mean	SD	Mean	SD	U	p
Score grades	8.13	1.77	8.68	0.81	-3.180	0.001*
Improvement	2.31	1.98	3.40	1.76	-4.953	0.000*

* Results are significantly different at p < 0.001.

Finally, we analyzed the students’ level of satisfaction according to the survey data. A total of 153 experimental group students completed the survey (about 89%). In general terms, the students positively evaluated the competitive experience (with an average score of 30.86 out of 50). The students liked learning through the participation in contests and they were motivated to find information in books or Internet to improve their positions in the ranking (3.22 and 3.59 out of 5, respectively). Moreover, despite the competitive nature of the QUESTOURnament tool, most students (70%) believed that this activity facilitated their relationship with other students.

On the other hand, using Pearson’s Correlation Coefficient, the results indicated that there was significant correlation between the students’ level of satisfaction with the competitive experience and their academic improvement, but this relation was weak (r = 0.292, p < 0.01).

To conclude, we analyzed which itinerary the students would recommend to other students. Most of students of the experimental group (88%) would recommend the itinerary B (competitive learning with the QUESTOURnament system). What was more interesting was that 54% students that had chosen the itinerary A would recommended the itinerary B due to the opinions of their classmates and the good atmosphere perceived in class.

## Discussion and conclusion

This paper reports on an experiment conducted to study the effects of the use of competitive learning methods in a medical course. We obtained some interesting conclusions: first, the students liked the QUESTOURnament tool since they regarded it as useful, motivating to participate more actively in their learning process and facilitating the learning and the relationship with other students.

During the Human Immunology course, the students felt a collaborative ambience within the classrooms, because they shared or debated about the different challenges. Although, medical students tend to be competitive [13, 28], this activity fostered classroom relationships (according to the data of satisfaction survey).

Moreover, the results of this study indicated that the use of the QUESTOURnament tool had favorable effects on the students’ academic outcomes. The students who used the tool obtained better final grades; in addition, the increasing academic improvement was significantly higher than that of the control group. As expected, there was a correlation, although weak, between the students’ satisfaction level with this method and the obtained score. Therefore, the presented results suggested that the QUESTOURnament tool could support effective learning strategies based on competition in medical students.

The positive results obtained in this study are in concordance with other similar studies about the role of competition in medical studies. Learning with competition improves the students’ academic results [19, 21, 28] and is considered enjoyable and engaging by them [20]. Thus, our findings are consistent with the previous studies reporting significant improvement in the competitive learning-driven students’ acquisition of knowledge and a greater satisfaction with the learning process. In addition, our findings report that well-designed competitive learning activities may foster the cooperation among students and provide a pleasant classroom environment.

The assayed active learning method–based on the competitive proposal of challenges and the answering of classmates’ ones–has worked very nicely because it promoted a collaborative classroom ambience, with the students debating about the correct answers of a given challenge. The students choosing the experimental group thus, had to study in a daily basis, either to propose challenges or to answer those proposed by their classmates. This fact could have contributed to a better grading of this group in Human Immunology, but also in a higher difference compared to the grades obtained in related courses during the first year of Medicine. Therefore, competitive strategies can be designed to get a more uniform distribution of knowledge sessions over time and, therefore, promoting learners’ deep understanding and long-term retention [29].

Students of the itinerary B (experimental group) followed a learning approach more according to the model of “constructive alignment” [30]. The learning outcomes of Human Immunology are divided into two groups or categories: “know” and “know how to do”. Students of itinerary B could demonstrate better whether they had achieved the intended learning outcomes (ILOs), since they faced different assessment methods. They solved, proposed and evaluated challenges, which might be very different in nature and thus, they could address different ILOs. Besides, each of the four contests corresponded to a different lesson and one criteria to evaluate challenges was their relevance to the topic of the lesson. Therefore, challenges in the different contests had to be aligned with the ILOs of the lessons. Moreover, they acquired a deeper learning of the “know how to do” part because, when proposing and solving challenges, they had to extrapolate which has been learnt to new scenarios.

This study had some limitations too. We did not make a random assignment to the control and experimental groups; because the two groups are nonequivalent, selection bias may exist. It is possible that students who volunteered to participate in the competitive itinerary were the most innately competitive. A more complete evaluation of cognitive learning styles would help to demonstrate more firmly the validity of the results. Besides, the experimental and control groups were not completely homogenous as the students in the experimental group had lower scores in previous courses than the students in the control group. Therefore, the greater improvement in course grades in the experimental group could be influenced by a lower starting grade and not only by the applied competitive strategy. The students who have a higher initial level of knowledge have a narrower range of improvement than classmates with a lower initial level and they may achieve less improvement [31]. Anyway, Shadish et al. [32] establish that when the pretest-posttest trend lines of a quasi-experiment cross over, it could be postulated that the posttest mean of the experimental group is amplified because it is easier to increase when the starting point is lower; however, this one can explain the amplification of an effect but not the creation of a totally artificial effect. Thus, though that is not the most desirable outcome, this pattern shows evidence of the effectiveness of the system within the non-equivalent groups design [33].

On the other hand, it is extremely difficult to create two homogeneous groups in experimental design, since there may be known factors (age, sex…) that can affect outcomes but are not of primary interest. In fact, a randomized blocks experimental design should have been made, instead a completely randomized design, in order to guarantee the existence of homogeneous groups and reduce noise or variance in the data [34].

Moreover, the non-equivalent groups design is probably the most frequently used design in social research [34]. For example, Zhang et al. [35] did a meta-analysis about the effectiveness of problem-based learning in medical courses, where they analyzed 31 studies from 2005 until 2014. They found that only two studies described an appropriate randomization process.

In this study, a randomized experimental design was not done because of ethical issues, which, as we have already mentioned, generates validity problems. In future studies, we could minimize this effect with a quasi-experimental design that uses a double pretest or switching replications. These approaches are less likely to permit causal interpretations of observed associations, according to Harris et al. [36], who presented an interesting review of 34 quasi-experimental studies in medical informatics, classifying them into several categories and analyzing the benefits and limitations of each approach.

Finally, we would like to conduct new future experiments to study the balance between speed and accuracy when answering the challenges. Pusic el al. [22] have argued that chronometry has been underutilized in the learning of medical procedures, being very useful for instruction designs as well as for assessment methods. Besides, they have stated that chronometry can increase motivation and therefore maximize learning, but also that it can be dangerous if it is wrongly implemented. There is a need to balance accuracy with speed. Our competitive tool -QUESTOURnament system- allows teachers to define simultaneously scoring and timing parameters to get the desired balance. It gives students feedback about scoring and timing, increasing the challenge level and enhancing self-regulation of learning [22]. Future experiments would allow us to define different strategies to get the balance between accuracy and speed according to the learning context, the maturity of the students or their current level of knowledge. Moreover, according to results obtained by Lei et al. [19] about team-competition, we are planning to use it within QUESTOURnament tool, paralleling the competitive learning among groups and the cooperation between students in the same group during the Human Immunology course.

## Supporting information

S1 Appendix Survey for measuring student satisfaction and collecting general data. (PDF)

## References

[1] Bhattacharya S, Nath S. Intelligent e-Learning Systems: An Educational Paradigm Shift. Int J Interact Multimed Artif Intelligence 2016;4(2):83–8.

[2] Verdú E, Regueras LM, Gal E, de Castro JP, Verdú MJ, Kohen-Vacs D. Integration of an intelligent tutoring system in a course of computer network design. Educ Technol Research Dev 2017;65(3):653–77.

[3] Alonso V, Arranz O. Big Data & eLearning: A Binomial to the Future of the Knowledge Society. Int J Interact Multimed Artif Intelligence 2016;3(6):29–33.

[4] Dolmans DHJM, Schmidt HG. What Do We Know About Cognitive and Motivational Effects of Small Group Tutorials in Problem-Based Learning?. Adv Health Sci Educ 2006;11(4):321–36. 10.1007/s10459-006-9012-8 16953462

[5] Visschers-Pleijers AJSF, Dolmans DHJM, Wolfhagen HAP, van der Vleuten CPM. Exploration of a method to analyze group interactions in problem-based learning. Med Teach 2004;26(5):471–8. doi: 10.1080/01421590410001679064 15369889 10.1080/01421590410001679064

[6] Dochy F, Segers M, van den Bossche P, Gijbels D. Effects of problem-based learning: a meta-analysis. Learn Instruct 2003;13(5):533–68.

[7] Colliver JA. Effectiveness of problem-based learning curricula: research and theory. Acad Med 2000;75(3):259–66. 10724315 10.1097/00001888-200003000-00017

[8] Albanese M. Problem-based learning: why curricula are likely to show little effect on knowledge and clinical skills. Med Educ 2000;34(9):729–38. 10972751 10.1046/j.1365-2923.2000.00753.x

[9] Norman G, Schmidt H. Effectiveness of Problem-Based Learning Curricula: Theory, Practice and Paper Darts. Med Educ 2000;3:721–8. 10.1046/j.1365-2923.2000.00749.x 10972750

[10] Hwang G-J, Wu P-H, Chen C-C. An online game approach for improving students’ learning performance in web-based problem-solving activities. Comput Educ 2012;59(4):1246–56.

[11] Verdú E, Regueras LM, Verdú MJ, Leal JP, de Castro JP, Queirós R. A distributed system for learning programming on-line. Comput Educ 2012;58(1):1–10.

[12] Anderson JR. On Cooperative and Competitive Learning in the Management Classroom. Mt Plains J Bus Econ 2006;7:1–10.

[13] Lempp H, Seale C. The hidden curriculum in undergraduate medical education: qualitative study of medical students’ perceptions of teaching. BMJ 2004;329:770–3. doi: 10.1136/bmj.329.7469.770 15459051 10.1136/bmj.329.7469.770 PMC520997

[14] Johnson D, Johnson R, Smith K. Cooperative learning and individual student achievement in secondary schools In: Pedersen JE, editor. Secondary schools and cooperative learning: theories, models, and strategies. New York: Garland Publishing; 1995 p. 3–54.

[15] Vandercruysse S, Vandewaetere M, Cornillie F, Clarebout G. Competition and students’ perceptions in a game-based language learning environment. Educ Technol Research Dev 2013 10 26;61(6):927–50.

[16] Regueras LM, Verdú E, Muñoz MF, Pérez MA, de Castro JP, Verdú MJ. Effects of Competitive E-learning Tools on Higher Education Students: A Case Study. IEEE Trans Educ 2009;52(2):279–85.

[17] Okereke C, Ugwuegbulam CN. Effects of Competitive Learning Strategy on Secondary School Students Learning Outcomes: Implications for Counselling. Int J Acad Res Prog Educ Dev 2014;3(2):137–43.

[18] Chen Z-H, Chen SY. When educational agents meet surrogate competition: Impacts of competitive educational agents on students’ motivation and performance. Comput Educ 2014;75:274–81.

[19] Lei J-H, Guo Y-J, Qiu Y-Y, Gong G-Z, He Y. Problem/case-based learning with competition introduced in severe infection education: an exploratory study. SpringerPlus 2016;5(1):1821 doi: 10.1186/s40064-016-3532-3 27818859 10.1186/s40064-016-3532-3 PMC5074983

[20] Janssen A, Shaw T, Goodyear P, Kerfoot BP, Bryce D. A little healthy competition: using mixed methods to pilot a team-based digital game for boosting medical student engagement with anatomy and histology content. BMC Med Educ 2015 10 12;15:173 doi: 10.1186/s12909-015-0455-6 26459198 10.1186/s12909-015-0455-6 PMC4603990

[21] Van Nuland SE, Roach VA, Wilson TD, Belliveau DJ. Head to head: The role of academic competition in undergraduate anatomical education. Anat Sci Educ 2015 9 1;8(5):404–12. doi: 10.1002/ase.1498 25319077 10.1002/ase.1498

[22] Pusic MV, Brydges R, Kessler D, Szyld D, Nachbar M, Kalet A. What’s your best time? Chronometry in the learning of medical procedures. Med Educ 2014 5 1;48(5):479–88. doi: 10.1111/medu.12395 24712933 10.1111/medu.12395

[23] Verdú E, Regueras L, Verdú MJ, Pérez MÁ, de Castro JP. QUEST: A Contest-Based Approach to Technology-Enhanced Active Learning in Higher Education. In: Impedovo S, Kalpic D, Stjepanovic Z, editors. Proceedings of 6th WSEAS International Conference on Distance Learning and Web Engineering. Lisboa (Portugal): WSEAS; 2006. p. 10–5.

[24] Bures EM, Abrami PC, Amundsen C. Student motivation to learn via computer conferencing. Res High Educ 2000;41(5):593–621.

[25] Chaput de Saintonge DM, Dunn DM. Gender and achievement in clinical medical students: a path analysis. Med Educ 2001;35(11):1024–33. 11703638 10.1046/j.1365-2923.2001.01043.x

[26] Budakoglu I, Erdemli E, Babadogan C. Learning styles of term 1 medical students in Turkish and English departments of medical faculty. Proc Soc Behavioral Sci 2011;00:000–000.

[27] Grasha AF. Teaching with Style—A practical guide to enhancing learning by understanding teaching & learning styles. Pittsburgh, PA: Alliance Publishers; 2012.

[28] Martínez-Bernal J, Sanabria-Rodríguez LB, López-Vagas O. Relationships between learning achievement, self-monitoring, cognitive style, and learning style in medical students. Praxis & Saber 2016 7 8;7(14):141–64.

[29] Panzarasa P, Kujawski B, Hammond EJ, Roberts CM. Temporal patterns and dynamics of e-learning usage in medical education. Educ Tech Research Dev 2016 2 1;64(1):13–35.

[30] Biggs JB. Enhancing teaching through constructive alignment. High Educ 1996;32(3):347–64.

[31] Lund JL, Kirk MF. Performance-Based Assessment for Middle and High School Physical Education. Champaign: Human Kinetics; 2010.

[32] Shadish WR, Cook TD, Campbell DT. Experimental and quasi-experimental designs for generalized causal inference. Boston: Houghton Mifflin; 2002.

[33] Trochim WMK, Donnelly JP. The Research Methods Knowledge Base Atomic Dog; 2006.

[34] Trochim WMK, Donnelly JP, Arora K. Research Methods: The Essential Knowledge Base Cengage Learning, 2nd Ed; 2015.

[35] Zhang Y, Zhou L, Liu X, Liu L, Wu Y, Zhao Z, et al The Effectiveness of the Problem-Based Learning Teaching Model for Use in Introductory Chinese Undergraduate Medical Courses: A Systematic Review and Meta-Analysis. PLoS ONE 2015;10(3): e0120884 https://doi.org/10.1371/journal.pone.0120884. 25822653 10.1371/journal.pone.0120884 PMC4378971

[36] Harris AD, McGregor JC, Perencevich EN, Furuno JP, Zhu J, Peterson DE, Finkelstein J. The Use and Interpretation of Quasi-Experimental Studies in Medical Informatics. J Am Med Inform Assoc 2006;13(1):16–23. doi: 10.1197/jamia.M1749 16221933 10.1197/jamia.M1749 PMC1380192

