# Supplementary material for: Effects of competitive learning tools on medical students: A case study
Source: PLoS One. 2018 Mar 8;13(3):e0194096. doi: 10.1371/journal.pone.0194096 (PMC5843339; doi:10.1371/journal.pone.0194096)
Supplement: S1 Appendix — (PDF) [file pone.0194096.s001.pdf]

Survey – QUESTOURnament tool

Part I: General information

Gender:

☐ Female

☐ Male

Age:

☐ <19 years

☐ 19-20 years

☐ 21-22 years

☐ > 23years

Group:

☐ Itinerary A

☐ Itinerary B

Select the options that best define you as a student (**Profile**)

☐ Competitive

☐ Collaborative

☐ Independent

☐ Contributive

Do you usually attend classes? (**Level of class attendance**)

(Value yourself between 0 = nothing, and 10 = totally)

Do you usually bring the subjects up to date? (**Level of hard-working**)

(Value yourself between 0 = nothing, and 10 = totally)

What itinerary would you recommend?

□ Itinerary A

□ Itinerary B

□ I don't know

Part II: Students' satisfaction and motivation (only students of itinerary B)

1 "Strongly Disagree"

2 "Moderately Disagree"

3 "Neutral"

4 "Moderately Agree"

5 "Strongly Agree"

	**1**	**2**	**3**	**4**	**5**
I like to learn the subject through the participation in the contest					
The contests have allowed me to deepen my knowledge and better understand the subject					
I assimilate the concepts better with this type of activities than with the traditional class					
Answering to the proposed challenges gives me satisfaction					
The contests have been a motivation to study more					
This type of learning motivates me to participate more					

actively in my learning					
The contests improve the quality of the learning process					
I would like other courses to integrate this activity					
This type of activities favors my relationship with my classmates					
The QUESTOURnament tool is very useful					
